# Root tip regeneration: Yet another feather in FERONIA's cap

**DOI:** 10.1093/plcell/koaf154

**Published:** 2025-06-12

**Authors:** Leonard Blaschek

**Affiliations:** Assistant Features Editor, The Plant Cell, American Society of Plant Biologists; Department of Plant & Environmental Sciences, University of Copenhagen, Frederiksberg C 1871, Denmark

It is hard to escape the impression that FERONIA (FER) is everywhere. Loss of this receptor-like kinase leads to astonishingly diverse effects, including in pollen tube growth, cell shape, root architecture, abiotic and biotic stress resistance, and flowering time. Synthesizing the available evidence, Malivert and Hamant (2023) proposed a disturbed balance between cell wall integrity and turgor pressure as a unifying feature of these phenotypes, although it is not clear whether FER acts primarily in perception, wall responses, or regulation of water uptake (Malivert and Hamant 2023).

Now, Yanan Shen and colleagues (Shen et al. 2025) add yet another facet to the role of FER. They show that Arabidopsis (*A. thaliana*) root tip regeneration after resection, as measured by restoration of morphology and gravitropic response, is modulated by RALF33–FER signaling. RAPID ALKALINIZATION FACTORs (RALFs) are apoplastic signaling peptides known to play roles in plant responses to biotic and abiotic stresses. Among the 5 RALFs expressed in the root that were tested, only RALF33 was upregulated after root tip resection. Confirming its role in regeneration, *ralf33* CRISPR alleles showed reduced regeneration rates, while the external supply of RALF33 improved root tip regeneration. Corroborating FER as the responsible RALF33 receptor, *fer* loss-of-function mutants in 2 Arabidopsis ecotypes had increased root tip regeneration rates while showing no response to exogenous RALF33 application. FER autophosphorylation at Y648—a proxy for FER activity—was reduced after RALF33 treatment, suggesting that RALF33 inhibits FER signaling.

In investigating the signaling cascade downstream of FER, preliminary results led the authors to focus on TOPLESS and TOPLESS-RELATED (TPR) proteins (Du et al. 2016). TPR mutants showed various alterations in root tip regeneration rates, including a decrease in *tpr1* and an increase in *tpr4.* Both proteins interacted with FER, as confirmed by yeast-2-hybrid, GST pull-down, co-immunopurification, and split luciferase assays. Additionally, regeneration rates in *tpr4,* but not in *tpr1,* were unaffected by RALF33 treatment, indicating that TPR4 might act downstream of RALF33–FER. Indeed, 16 TPR4 residues could be phosphorylated by the FER kinase domain in an *E. coli* system. The TPR4^A^ variant, generated by mutating the 8 phosphorylated residues located in conserved stretches, precipitated higher regeneration rates than the wild-type protein. This suggests that TPR4 phosphorylation by FER is important for its role in wounding responses. The last step in this comprehensive investigation was to try to identify the transcription factor repressed by TPR4 in response to RALF33–FER signaling. ERF115 was an obvious candidate, given its importance in root tip regeneration (Heyman et al. 2016). Indeed, Shen et al. (2025) showed an interaction between TPR4 and ERF115, which was impaired by RALF33 treatment and in the TPR4^A^ variant. In addition, analysis of TPR4::GFP and TPR4^A^::GFP constructs indicated that their phosphorylation led to TPR4 translocation into the nucleus. The nuclear TPR4–ERF115 interaction, in turn, suppressed the activating effect of ERF115 on downstream targets, as shown by the example of the *PHYTOSULFOKINE 2 PRECURSOR 2* promoter.

Altogether, Shen et al. (2025) propose a link between RALF33 accumulation in response to wounding and derepression of ERF115 to induce root tip regeneration (Figure). Curiously, regeneration rates in the *erf115* mutant as shown by Shen et al. (2025) were not significantly different from the wild type, contrasting the results from Heyman et al. (2016). Similarly, the regeneration rates of *fer* mutants, while consistently higher than in the wild type, varied between 60% and >90% in different experiments (Shen et al. 2025). This variability highlights the presence of several additional regulators of regeneration, including the REF1–PORK1–WIND1 module (Yang et al. 2024) and jasmonate-responsive transcription factor ERF109 (Zhou et al. 2019). The RALF33–FER–TPR4–ERF115 signaling module described by Shen et al. (2025), however, provides a direct link from apoplastic signaling to transcriptional wounding responses, bringing us one step closer to understanding the regulation of root tip regeneration and mapping the functional extent of FER.

**Figure.**
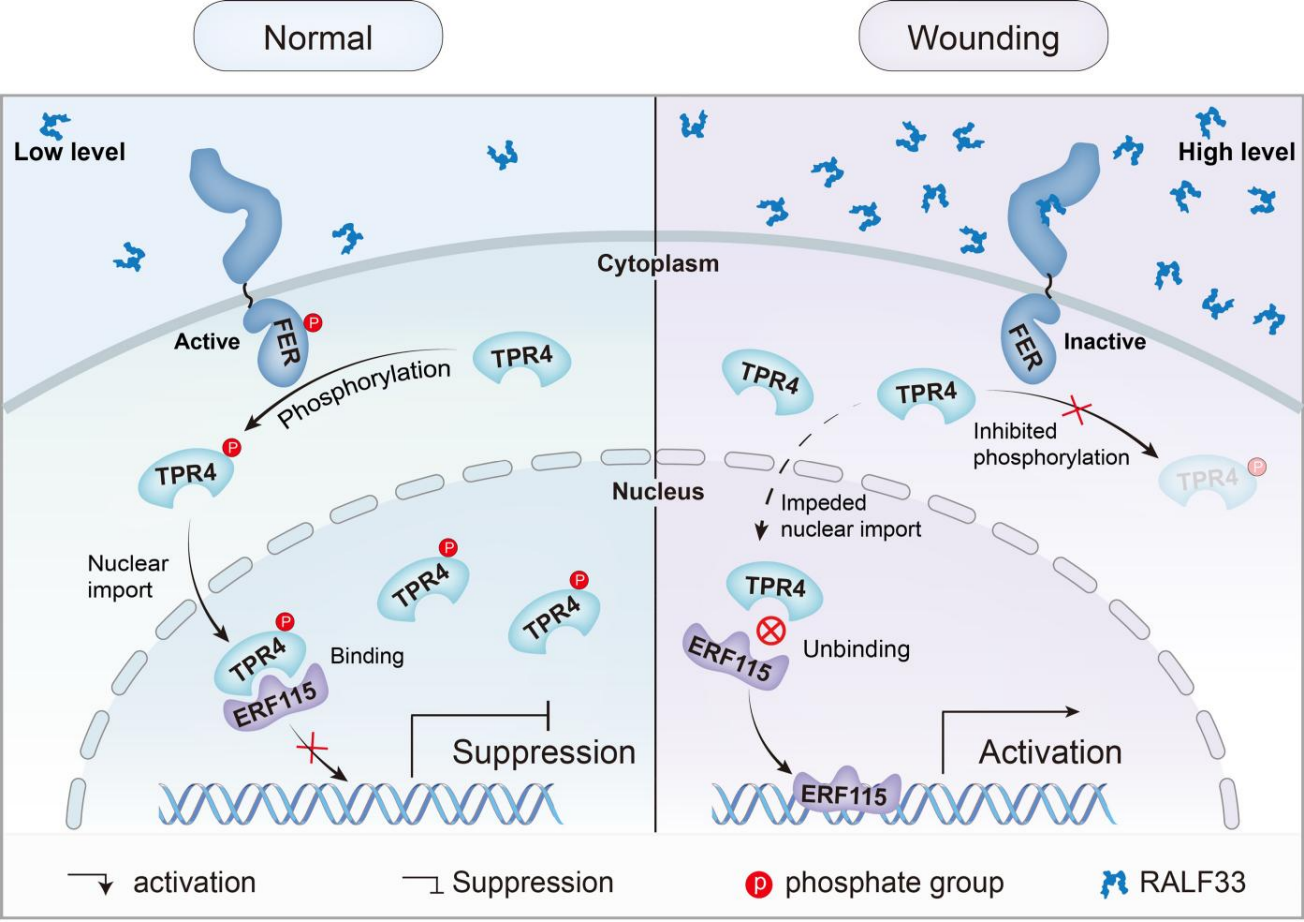
Proposed model of the RALF33–FER–TPR4–ERF115 signaling module. Reprinted from Shen et al. (2025), Figure 6.

## Recent related articles in *The Plant Cell*

- Wang et al. (2024) showed that FER, together with the transcription factor RD26, plays a role in plant immunity against the vascular tissue pathogen *Ralstonia solanacearum* by regulating lignin deposition
- Wang et al. (2022) provided integrated omics data to interrogate the multifaceted consequences of *fer* loss-of-function mutations
- Lee et al. (2024) described a novel transcription factor regulating jasmonate- and ERF109-mediated de novo root organogenesis in Arabidopsis

## Data Availability

No original data was generated for this *In Brief.*

## Dive Curated Terms

The following phenotypic, genotypic, and functional terms are of significance to the work described in this paper:

RALF33 Gramene: AT4G15800
RALF33 Araport: AT4G15800

TPR4 Gramene: AT3G15880
TPR4 Araport: AT3G15880

ERF115 Gramene: AT5G07310
ERF115 Araport: AT5G07310

TPR1 Gramene: AT1G80490
TPR1 Araport: AT1G80490

FER Gramene: AT3G51550
FER Araport: AT3G51550
